# A94 PRE-RESECTION OPTICAL EVALUATION RELIABLY DIFFERENTIATES BETWEEN SERRATED AND ADENOMATOUS LARGE NON-PEDUNCULATED COLORECTAL POLYPS

**DOI:** 10.1093/jcag/gwad061.094

**Published:** 2024-02-14

**Authors:** S X Jiang, A Zarrin, A Walia, C Galorport, W Xiong, R Enns, E Lam, N Shahidi

**Affiliations:** The University of British Columbia Faculty of Medicine, Vancouver, BC, Canada; The University of British Columbia Faculty of Medicine, Vancouver, BC, Canada; St. Paul’s Hospital, Division of Gastroenterology, University of British Columbia, Vancouver, BC, Canada; St. Paul’s Hospital, Division of Gastroenterology, University of British Columbia, Vancouver, BC, Canada; University of British Columbia, Department of Pathology and Laboratory Medicine, Vancouver, BC, Canada; St. Paul’s Hospital, Division of Gastroenterology, University of British Columbia, Vancouver, BC, Canada; St. Paul’s Hospital, Division of Gastroenterology, University of British Columbia, Vancouver, BC, Canada; St. Paul’s Hospital, Division of Gastroenterology, University of British Columbia, Vancouver, BC, Canada

## Abstract

**Background:**

Modality selection between cold snare resection (CSR) and endoscopic mucosal resection (EMR) or endoscopic submucosal dissection (ESD) is largely predicated on the ability to differentiate between serrated and adenomatous histopathology. While optical evaluation has modest accuracy for diminutive polyps, performance has not been evaluated for large non-pedunculated colorectal polyps (LNPCPs).

**Aims:**

To evaluate the performance of pre-resection optical evaluation to differentiate between serrated and adenomatous LNPCPs.

**Methods:**

Consecutive patients ampersand:003E 18 years of age who underwent endoscopic resection for a LNPCP were enrolled in a prospective single center observation cohort study (clinicaltrials.gov ID: NCT05402696). Pre-resection optical evaluation was performed using high-definition white-light and narrow-band imaging (NBI) with or without near-focus. The Japanese NBI Expert Team (JNET) classification was used to differentiate between serrated (JNET I) vs. adenomatous (JNET IIA, IIB) LNPCPs. Traditional serrated adenomas (TSAs) and cancers were excluded from analysis. Sensitivity, specificity, and accuracy were used to evaluate optical evaluation performance.

**Results:**

From 06/2022-09/2023, 266 patients underwent 282 procedures for a total of 335 LNPCPs. Median size was 30mm (IQR 20-40mm). Histopathology identified 215 (64.2%) adenomatous, 91 (27.2%) serrated, 16 (4.8%) cancerous, and 13 (3.9%) other LNPCPs; including 5 TSAs. Of the 91 serrated lesions, 90 (98.9%) were predicted as serrated; sensitivity, specificity, and accuracy were 98.90% (95% CI 94.03-99.97), 99.53% (95% CI 97.44-99.99), 99.35% (95% CI 97.66-99.92), respectively. Of the 215 adenomatous lesions, 213 (99.1%) were predicted as adenomatous; sensitivity, specificity, and accuracy were 99.07% (95% CI 96.68-99.89), 98.90% (95% CI 94.03-99.97), 99.02% (95% CI 97.16-99.80), respectively.

**Conclusions:**

Optical evaluation demonstrates excellent performance characteristics to differentiate between serrated and adenomatous LNPCPs; therefore, empowering endoscopists to reliably apply a selective resection algorithm between CSR, EMR and ESD.

**Funding Agencies:**

None

